# Comparison of Patency Between Sequential and Individual Radial Artery Anastomoses in Coronary Artery Bypass Grafting

**DOI:** 10.31083/RCM39689

**Published:** 2025-11-12

**Authors:** Siyu Zhang, Chunyuan Wang, Zhan Hu, Yan Zhang, Zhe Zheng

**Affiliations:** ^1^Department of Cardiovascular Surgery, Chinese Academy of Medical Sciences and Peking Union Medical College Fuwai Hospital, 100037 Beijing, China; ^2^National Health Commission Key Laboratory of Cardiovascular Regenerative Medicine, Central China Fuwai Hospital of Zhengzhou University, 451400 Zhengzhou, Henan, China

**Keywords:** coronary artery bypass, radial artery graft, sequential graft

## Abstract

**Background:**

Current evidence suggests that multiple arterial grafting improves long-term survival following coronary artery bypass grafting (CABG). Sequential radial artery (RA) grafting is known to be a safe approach for maximizing arterial revascularization. This study aimed to compare RA conduit patency between sequential and individual grafting.

**Methods:**

A total of 274 CABG patients who received at least one RA graft between January 2017 and June 2021 were included in our retrospective study. The occlusion of RA anastomoses was assessed by postoperative computed tomography angiography or coronary angiography at follow-up. Group comparisons for long-term outcomes were conducted using adjusted Cox regression models.

**Results:**

The median image follow-up time was 1.8 years. Among the 278 total RA grafts, 208 were individual and 70 were sequential. Multivariable Cox analysis found that sequential anastomoses were an independent risk factor for RA graft occlusion (adjusted hazard ratio = 2.45, 95% confidence interval (CI): 1.15–5.22; *p* = 0.020). However, the observed rate of occlusion was low (2.9%) when all the target vessels for a sequential graft had a quantitative flow ratio of ≤0.71.

**Conclusions:**

Sequential RA grafting is associated with graft occlusion compared with individual grafting. For each RA graft, the selection of only one target vessel with significant functional stenosis may be preferable to achieve superior long-term patency.

## 1. Introduction

Several studies have confirmed that utilization of two or more arterial grafts is associated with better long-term survival compared to conventional single-arterial coronary artery bypass grafting (CABG) based on the left internal thoracic artery (LITA) and saphenous vein graft (SVG) [1, 2, 3]. Given its relatively easy harvest and lower risk of deep sternal infection compared to bilateral internal thoracic artery (ITA) [4], the radial artery (RA) has emerged as the predominant choice for a second arterial graft in both the United States and Europe [5, 6]. To improve long-term cardiac outcomes, the most recent guidelines have increased their recommendation level for employing RA grafts to class I [7].

Frequent use of the right RA for angiography prior to CABG has raised concerns about intimal tears and impending graft failure [8]. Therefore, unilateral RA grafting is often used more frequently than bilateral RA. Sequential grafting of the RA is a safe method of choice and helps to maximize the number of constructed arterial grafts. A number of studies have investigated the long-term outcomes of CABG with sequential RA grafting. These reported superior 10-year survival compared with conventional LITA and SVG [1, 9]. The RA conduit is known to be sensitive to competitive flow from the native coronary artery, which may be a key factor in premature graft loss [10]. However, multiple distal anastomoses in sequential grafts can increase the risk of coronary steal and graft flow reversal [11]. Previous studies did not account for significant heterogeneity in the target vessels of RA grafts, and hence the efficacy of sequential RA grafting remains unclear.

We previously reported that the preoperative quantitative flow ratio (QFR) of target vessels at the site of RA anastomoses was a robust predictor of occlusion [12]. Indeed, QFR proved to be a more accurate indicator of RA graft occlusion than the degree of stenosis. There is currently a lack of published literature on the different risk factors for RA graft patency. Therefore, the aim of this study was to conduct a risk-adjusted comparison of long-term patency between individual and sequential RA grafting.

## 2. Materials and Methods

### 2.1 Patients

This retrospective study was approved by the Ethics Committee of Fuwai Hospital (No. 2021-1554), which also waived the need for written informed consent.

The cohort included consecutive patients who received CABG procedures at Fuwai Hospital from January 2017 to June 2021. Patient inclusion criteria were as follows: (1) received angiography at our center within 1 year before surgery; (2) at least one RA graft was used. Patient exclusion criteria were: (1) lack of imaging follow-up for graft patency beyond three months postoperatively; (2) poor quality of angiographic images hindering QFR measurement.

To evaluate the patency of sequential grafting, all RA grafts were first categorized into either individual or sequential groups. To compare the clinical outcomes between these two groups, patients with both individual and sequential grafts were excluded.

### 2.2 Surgical Technique

Median sternotomy was employed for all patients. In every procedure, the left ITA was *in situ* for grafting to the left anterior descending (LAD), while the RA served as an additional arterial conduit, either as the second or third graft. The sole basis for selection of the target vessel was preoperative angiography. Radial arteries were harvested using conventional open techniques with pedicle, then bathed in a solution composed of papaverine, diltiazem, and warm saline with heparinized autologous blood prior to grafting. All procedures were performed by highly experienced surgeons who adhered to standardized protocols. On-pump surgery was performed with cardiopulmonary bypass under cardioplegic arrest. Individual RA grafts had only one distal end-side anastomosis, whereas sequential RA grafts were anastomosed to two or more target vessels. The RA graft was connected proximally to the ascending aorta through a direct anastomosis.

### 2.3 Data Collection

Preoperative coronary angiography was conducted using a conventional percutaneous approach via either the femoral or radial artery. The degree of stenosis in target vessels of RA grafts was visually estimated by two experienced operators. QFR computation was performed using AngioPlus Core software (V3.0, Pulse Medical Imaging Technology, Shanghai Co., Ltd., Shanghai, China) by two experienced analysts (**Supplementary Fig. 1**). In cases where QFR could not be measured due to total occlusion of the target vessel, an assigned default value of 0.50 was given, as described in a previous study [13]. All patients were recommended to receive regular coronary computed tomography angiography (CCTA) or angiography follow-up at three months, one year, and three years postoperatively, as well as follow-up through outpatient consultation or telephone interviews. The percentage of patients who were followed up by imaging at each time point is shown in **Supplementary Table 1**.

### 2.4 Study Endpoints

The primary endpoint was the occlusion of RA grafts, as assessed by CCTA or angiography at least three months after surgery. Occlusion of an RA graft was defined as total occlusion in any segment between the proximal and distal anastomoses of the graft. For sequential RA grafting, we aimed to position the distal anastomoses at the optimal target site with favorable run-off and a suitable degree of stenosis (>75%). Therefore, when assessing sequential bypass grafts, occlusion of even a single distal segment of the sequential graft was regarded as a sign of total occlusion, even if proximal segments of the graft were patent (**Supplementary Fig. 2**). The date when occlusion was initially documented by CCTA or angiography served as the time point for assessing graft occlusion. All postoperative CCTA and angiography images were independently reviewed by a radiologist at a central core laboratory and by a cardiac surgeon. Any discrepancies were resolved by involving another cardiac surgeon.

The secondary endpoint was major adverse cardiac or cerebrovascular events (MACCEs) in patients, defined as the composite outcome of all-cause mortality, myocardial infarction, stroke, or repeat revascularization.

### 2.5 Statistical Analysis

Depending on their distribution, continuous variables were presented as the mean ± standard deviation, or the median and interquartile range [IQR]. Categorical variables were presented as counts and percentages. Continuous variables were compared using the Student’s *t*-test or Mann-Whitney U test, while categorical variables were compared using the Chi-square test or Fisher’s exact test. Outcomes were reported as raw numbers and linearized event rates per 1000 patient-years. Cumulative incidences were also determined and are presented graphically.

A multivariable Cox proportional hazards model was used to evaluate the impact of the anastomoses method on graft outcomes. The proportional hazards assumption in the Cox model was assessed using Schoenfeld residuals. Variables were chosen based on previous literature and clinical plausibility. They included baseline characteristics and intraoperative conditions. The QFR of the target vessel was set at a threshold of 0.71 based on our previous findings [12]. For the primary outcome, subgroup and interaction-term analyses were used to investigate possible effect modifiers. These had been prespecified.

Statistical analyses were performed using R software, version 4.1.2 (R Foundation for Statistical Computing, Vienna, Austria). *p*-values were 2-sided, and *p*-values < 0.05 were considered statistically significant.

## 3. Results

### 3.1 Patients and Grafting Characteristics

**Fig. 1.**
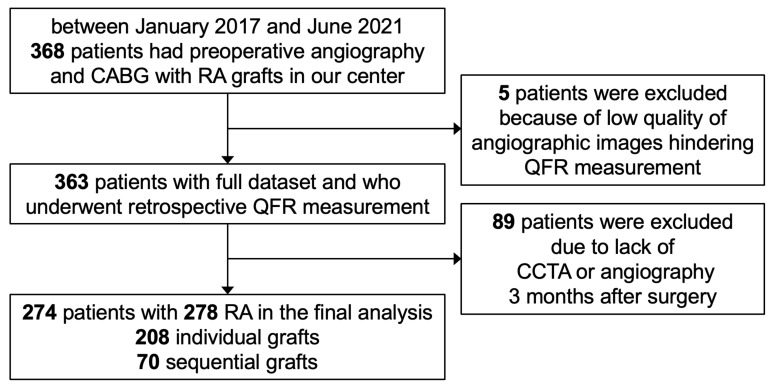
**Study flow chart**. CABG, coronary artery bypass grafting; RA, radial artery; QFR, quantitative flow ratio; CCTA, coronary computed tomography angiography.

A total of 368 patients underwent preoperative angiography and CABG with at least one RA graft between January 2017 and June 2021 in our center. Five patients were excluded from the QFR measurement due to poor quality of angiographic images. Of the remaining 363 patients, 89 were excluded due to a lack of imaging follow-up after three months postoperatively. Consequently, the final analysis included 274 patients with a total of 278 RA grafts (Fig. 1). The majority of patients (98.5%) underwent one single RA grafting, either as an individual or sequential method. Only 3 patients underwent two separate RA grafts using the individual method, while one patient had both an individual graft and a sequential graft.

The baseline demographic, clinical, and operative profiles of patients who underwent individual or sequential grafts are shown in Table 1. The average age of patients at the time of surgery was 53.8 ± 9.4 years, with 93.1% being males. While many baseline characteristics were comparable, significant differences were observed in the distribution of target vessels and in the proportion of on-pump versus off-pump procedures.

**Table 1.** **Comparison of patient characteristics between those undergoing individual or sequential radial artery grafts**.

	Individual (n = 208)	Sequential (n = 70)	*p*-value
Male	197 (94.7%)	62 (88.6%)	0.137
Age, years	53.4 ± 8.8	55.3 ± 11.1	0.147
BMI, kg/m^2^	26.2 ± 3.0	26.3 ± 2.8	0.843
EF <50%	11 (5.3%)	3 (4.3%)	0.987
Smoking	141 (67.8%)	43 (61.4%)	0.408
Diabetes	77 (37.0%)	27 (38.6%)	0.929
Hypertension	120 (57.7%)	46 (65.7%)	0.297
Hypercholesterolemia	170 (81.7%)	57 (81.4%)	>0.999
PVD	8 (3.8%)	6 (8.6%)	0.212
Prior PCI	43 (20.7%)	13 (18.6%)	0.836
Prior MI	60 (28.8%)	23 (32.9%)	0.629
3-vessel disease	129 (62.0%)	36 (51.4%)	0.156
Left main disease	12 (5.8%)	3 (4.3%)	0.865
Distal target diameter, mm	1.92 ± 0.45	1.89 ± 0.41	0.699
Distal target to left coronary system^*^	122 (58.7%)	60 (85.7%)	<0.001
On-pump	107 (51.4%)	12 (17.1%)	<0.001
Imaging follow-up, years	2.0 [1.2, 3.3]	1.4 [1.0, 2.2]	0.008
QFR of distal anastomoses	0.58 [0.50, 0.74]	0.54 [0.50, 0.73]	0.905
QFR >0.71	66 (31.7%)	24 (34.3)	0.805
DS of distal anastomoses, %	90.0 [80.0, 95.0]	90.0 [80.0, 99.0]	0.488

QFR, quantitative flow ratio; DS, degree of stenosis; BMI, body mass index; EF, ejection fraction; PVD, peripheral vascular disease; PCI, percutaneous coronary intervention; MI, myocardial infarction. *The left coronary system includes diagonal, ramus intermedius, obtuse marginal and posterior branches of left ventricle from circumflex.

The RA grafts included 70 sequential (61 double, 9 triple) and 208 individual configurations. Compared to the individual RA group, the sequential group had a lower proportion of on-pump CABG (17.1% vs. 51.4%, *p* < 0.001) and more distal anastomoses to the left coronary system, including diagonal, ramus intermedius, obtuse marginal and posterior branches of left ventricle from circumflex (85.7% vs. 58.7%, *p* < 0.001) (Table 1). There was no significant difference in the QFR of distal anastomoses between sequential and individual RA grafts, with a similar proportion of QFR >0.71 in both groups. Table 2 summarizes the coronary targets of RA grafts stratified by the grafting configuration. Individual grafts were most commonly anastomosed to the right coronary system (40.9%), followed by the circumflex system (36.5%). The most common sequence for sequential grafts was the diagonal/ramus intermedius and circumflex system (67.1%).

**Table 2.** **Distribution of coronary anastomoses for RA grafts**.

Coronary target		Individual (n = 208)	Sequential (n = 70)
Single coronary system			
	Diagonal or ramus intermedius	47 (22.6%)	4 (5.7%)
	Circumflex	76 (36.5%)	9 (12.9%)
	RCA	85 (40.9%)	5 (7.1%)
Multiple coronary systems			
	Diagnal/Ramus + Circumflex	-	47 (67.1%)
	Circumflex + RCA	-	5 (7.1%)

Circumflex system includes obtuse marginal and posterior branches of left ventricle from circumflex; RCA, right coronary artery system (includes right main, acute marginal, posterior descending, and posterior branches of left ventricle from right coronary); RA, radial artery.

### 3.2 Outcomes

**Fig. 2.**
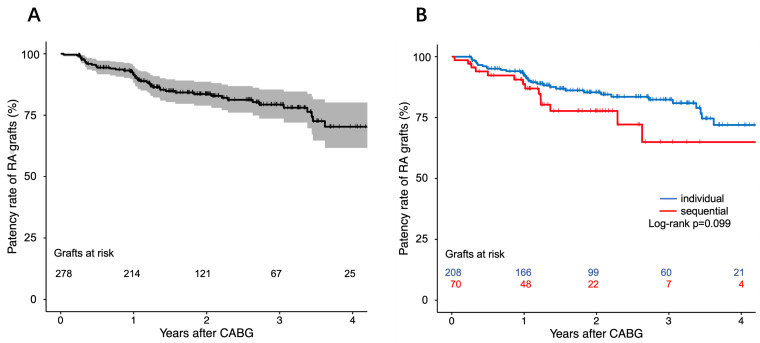
**Kaplan-Meier curve of RA grafts patency**. (A) Kaplan-Meier curve of total RA grafts patency. (B) Comparison of cumulative RA grafts patency between individual and sequential groups. RA, radial artery; CABG, coronary artery bypass grafting.

No perioperative deaths occurred in the study cohort. The median duration of imaging follow-up was 1.8 years (IQR [1.1, 2.9]), during which the overall unadjusted RA graft patency reached 82.0%. Kaplan-Meier analysis revealed that cumulative patency rates at 1, 2, and 3 years were 91.5%, 83.6%, and 79.3%, respectively (Fig. 2A). The incidence rate for graft occlusion was 79 events per 1000 patient-years in the individual group, versus 136 events per 1000 patient-years in the sequential group (Table 3). Although log-rank analysis showed no statistically significant difference between the two groups (*p *= 0.099; Fig. 2B), sequential grafting was identified as an independent risk factor for graft occlusion in multivariable analysis (adjusted hazard ratio (aHR) = 2.45; 95% CI: 1.15–5.22; *p* = 0.020) (Table 4). QFR >0.71 was also identified as an independent risk factor for RA graft occlusion, whereas prior myocardial infarction was a protective factor against graft occlusion. Subgroup analyses showed no significant interactions between treatment effect and patient characteristics, target vessel QFR, target location, or surgical technique (**Supplementary Fig. 3**).

**Table 3.** **Outcomes for the individual and sequential groups**.

Outcomes	Individual (n = 208)		Sequential (n = 70)		Treatment effect*			
	No. of events	E/kPY	No. of events	E/kPY	HR (95% CI)	*p*-value	aHR (95% CI)	*p*-value
Graft occlusion	35 (16.8%)	79	15 (21.4%)	136	1.67 (0.90–3.08)	0.103	2.45 (1.15–5.22)	0.020
MACCEs^#^	10/204 (4.9%)	24	1/69 (1.4%)	9	0.39 (0.05–3.02)	0.364	0.14 (0.01–1.63)	0.117

*Results are from a multivariable Cox regression model, with the individual group used as the reference group. ^#^The analysis of MACCEs excluded 1 patient who received both individual and sequential RA grafts. E/kPY, Events per 1000 Patient-Years; aHR, adjusted hazard ratio; 95% CI, 95% confidence interval; MACCEs, major adverse cardiac or cerebrovascular events; HR, hazard ratio.

**Table 4.** **Univariate and multivariate analysis of risk factors for the occlusion of radial artery grafts**.

Variable	HR (univariate)	*p*-value	aHR (multivariate)	*p*-value
Sequential graft	1.67 (0.90–3.08)	0.103	2.45 (1.15–5.22)	0.020
QFR >0.71	9.66 (4.82–19.34)	<0.01	10.55 (4.72–23.56)	<0.01
Age >60 years	0.45 (0.19–1.06)	0.066	0.58 (0.23–1.44)	0.239
Female	0.70 (0.17–2.91)	0.627	1.05 (0.24–4.61)	0.951
BMI	1.05 (0.95–1.15)	0.350	0.97 (0.85–1.10)	0.630
EF <50%	0.81 (0.20–3.35)	0.773	-	-
Diabetes	1.23 (0.70–2.16)	0.467	-	-
Hypertension	1.12 (0.63–1.99)	0.692	-	-
Hypercholesterolemia	0.92 (0.44–1.89)	0.815	-	-
PVD	1.16 (0.28–4.80)	0.837	-	-
Prior MI	0.50 (0.23–1.10)	0.085	0.40 (0.18–0.87)	0.021
Smoking	1.20 (0.65–2.24)	0.559	-	-
On-pump surgery	0.94 (0.51–1.73)	0.838	0.94 (0.47–1.90)	0.877
Target on left system	1.00 (0.56–1.79)	0.797	1.51 (0.66–3.34)	0.309

HR, hazard ratio; aHR, adjusted hazard ratio; QFR, quantitative flow ratio; BMI, body mass index; EF, ejection fraction; PVD, peripheral vascular disease; MI, myocardial infarction.

After a median follow-up period of 1.8 years (IQR [0.9, 3.0]), the overall incidence of MACCEs was just 4.0%. All patients survived without experiencing a myocardial infarction. Eight patients suffered a stroke, and 3 patients underwent percutaneous coronary intervention. The Kaplan-Meier curve for patient outcomes is shown in **Supplementary Fig. 4**. After the exclusion of one patient who received both individual and sequential RA grafts, the incidence of MACCEs was not significantly different between the individual (4.9%) and sequential (1.4%) groups (aHR = 0.14, 95% CI: 0.01–1.63, *p* = 0.117) (**Supplementary Table 2**).

### 3.3 QFR of Sequential Anastomoses and Patency of Grafts 

**Fig. 3.**
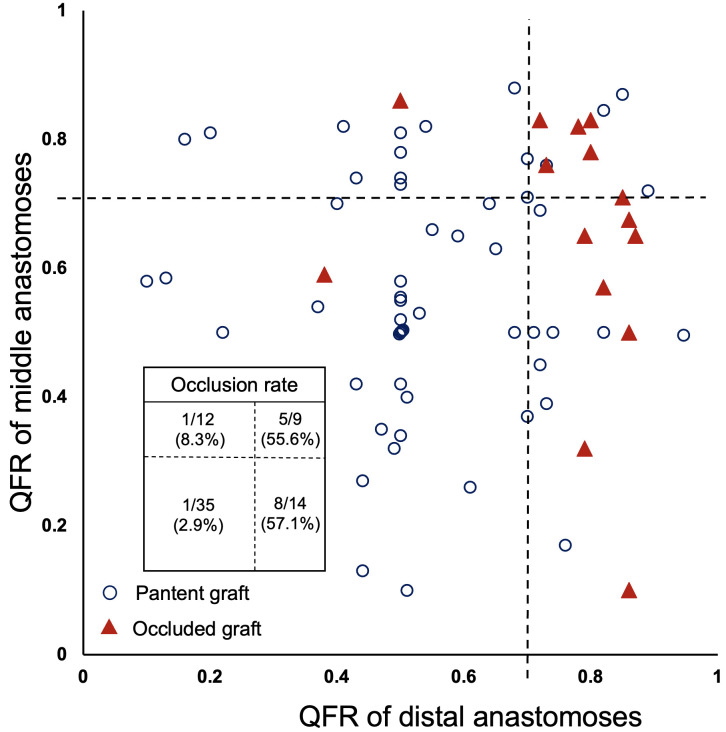
**The QFR of target vessels in sequential grafts and graft patency**. Each point represents an RA graft. The x-axis shows the QFR of the distal anastomoses of sequential grafts, while the y-axis shows the QFR of the middle anastomoses (average used when multiple). QFR, quantitative flow ratio; RA, radial artery.

The sequential grafts were classified into four categories based on the QFR of distal and middle anastomoses, with a cut-off value of 0.71. The graft occlusion in each group is shown in Fig. 3. When the QFR of both the distal and middle anastomoses was ≤0.71, the incidence of graft occlusion was only 2.9% (1 out of 35 grafts). When the QFR of distal anastomoses was ≤0.71 and the QFR of middle anastomoses was >0.71, the incidence of occlusion increased to 8.3% (1 out of 12 grafts). Moreover, when the QFR of distal anastomoses was >0.71 and that of middle anastomoses was ≤0.71, the incidence of occlusion increased to 57.1% (8 out of 14 grafts). All of the 8 grafts occluded in this subgroup were observed in the distal segment, with the proximal segment remaining patent.

## 4. Discussion

This single-center retrospective cohort study investigated the long-term patency of RA bypass grafts to identify risk factors associated with graft occlusion. The sequential method of anastomoses was found to be a significant predictive factor for RA graft occlusion, as well as target vessels with a QFR >0.71.

Following revival of the RA conduit in the 1990s [14], its utilization in CABG has been supported by solid evidence demonstrating its clinical benefits. The RADIAL project found that patients with RA grafts as the second conduit in addition to the left ITA had significantly reduced incidence of MACCEs at both 5- and 10-year follow-up compared to SVG [2, 3]. The RA also showed advantages over the right ITA, with better long-term patency and fewer sternal-related complications [15]. Consequently, utilization of the RA was recommended in the guidelines and is frequently utilized in clinical practice as the primary choice for second arterial graft material [7, 16].

Sequential RA grafting helps to achieve complete revascularization and maximize the number of arterial grafts. However, there have been only a few studies on the patency of sequential RA grafting. Schwann *et al*. [9] assessed the angiographic results of sequential RA grafting in 122 patients with a median follow-up of 1.8 years, finding an overall patency of 71%. Furthermore, no significant difference in patency was observed between sequential and individual RA grafts. However, the conclusion reached by these authors is open to question, first because the angiographic follow-up was symptom-driven, and second because the sample size of nonsequential grafts in their study was limited. There are also ongoing concerns about the long-term patency of sequential RA grafts due to the coronary steal phenomenon.

Clinical and demographic characteristics were taken into account in the present study, and specifically the functional characteristics of target vessels. We observed a significant association between the sequential grafting method and the occlusion of RA grafts following CABG surgery. Several pathophysiological factors may be responsible for the superior patency observed with single conduits. First, sequential grafts may be more susceptible to blood flow reversal compared to individual grafts. In the context of RA to non-LAD bypass, Nakajima *et al*. [17] reported that 86.3% of competitive flow occurred at the distal end of sequential grafts. Graft occlusion in our cohort was lowest when the QFR of both the middle and distal anastomoses was ≤0.71. However, the occlusion of sequential grafts increased when the QFR of any anastomoses was >0.71. Similar to the SVG sequential bypass technique [18], our strategy was aimed at targeting the largest lesion with the highest degree of stenosis, and with good run-off as the most distal anastomoses. Selection of the intermediate target may affect long-term patency of the entire sequential grafts. Second, incisions made in the middle of RA conduits may lead to additional endothelial damage and the occurrence of RA spasm. Subsequent focal neointimal hyperplasia at anastomotic sites could potentially affect the patency of the graft. Third, sequential bypass grafts carry a greater risk of kinking and positioning complications. In the present study, the majority (82.9%) of sequential grafts were anastomosed using the off-pump method, thereby mitigating potential errors associated with the measurement of graft length. Moreover, we adjusted for the use of cardiopulmonary bypass and observed no significant effect of this procedure on graft patency. Further research is warranted to address this issue in more depth.

Our results may provide an explanation for the unsuitability of extensive constructions involving more than two arterial conduits using sequential RA grafting. In a meta-analysis of 10,287 patients, Gaudino *et al*. [19] found that the use of a third individual arterial graft was associated with a 24% relative survival benefit. In contrast, Schwann *et al*. [20] did not observe a statistically significant advantage in 15-year survival in patients with three-artery grafts compared to those with two-artery grafts. The latter study consisted almost entirely of patients who received a left ITA and supplemental sequential RA grafts (bilateral, sequential, or both) rather than bilateral ITA. The construction of an extensive artery bypass based on sequential RA may be less effective clinically compared to the use of bilateral ITA and an individual RA. The higher attrition rate of sequential RA grafts observed in our study may be due to one of these underlying factors.

We also found that prior myocardial infarction was a protective factor for RA graft patency. This observation concurs with the results of subgroup analysis in the RADIAL project, which favored RA over SVG [2]. We speculate that patients who had collateral flow were prone to limited previous infarct size, and also had enhanced native competitive flow, thereby leading to decreased patency of RA grafts after CABG [21].

These findings underscore the potential clinical utility of QFR in guiding arterial graft planning during CABG. Preoperative QFR assessment may help identify target vessels with high competitive flow risk, particularly in sequential grafting, and thus assist in selecting optimal anastomosis sites to reduce the likelihood of graft occlusion. Incorporating QFR into routine surgical planning may enhance long-term graft patency and improve patient outcomes, although prospective validation is warranted.

This study has several limitations. First, its retrospective design may have included unmeasured confounding factors. Baseline differences, such as less use of cardiopulmonary bypass in the individual group, may also have introduced some bias in patency comparisons, as the choice of on-pump or off-pump grafting was based on coronary anatomy and surgical judgment. However, all procedures were performed by highly experienced surgeons who adhered to standardized protocols. Additionally, sequential anastomosis remained as an independent risk factor for occlusion after the incorporation of variables into the multivariate model. Second, some patient selection bias for RA grafting may have occurred. However, our imaging follow-up was planned and not symptom-driven, and up to 75.4% of patients successfully completed postoperative imaging follow-up. Follow-up of incomplete and non-uniform imaging may introduce bias, despite the use of time-to-event analyses that account for censoring. Third, the limited sample size and relatively short follow-up gave rise to a low incidence of MACCEs, particularly in the sequential group, making it difficult to assess the impact of RA occlusion on clinical outcomes.

## 5. Conclusions

Compared to individual RA bypass grafting, sequential RA was associated with inferior graft patency after CABG. Multiple target options exist for sequential grafts, and inappropriate selection may result in increased competition of blood flow and subsequent failure of the RA graft.

## Data Availability

The data underlying this article cannot be shared publicly due to the policy of data sharing of our centre. The data will be shared on reasonable request to the corresponding author.

## Abbreviations

CABG, coronary artery bypass grafting; RA, radial artery; ITA, internal thoracic artery; SVG, saphenous vein graft; QFR, quantitative flow ratio; CCTA, coronary computed tomography angiography; MACCEs, major adverse cardiac or cerebrovascular events; IQR, interquartile range; LAD, left anterior descending; 95% CI, 95% confidence interval; aHR, adjusted hazard ratio.

## Supplementary Material

Supplementary material associated with this article can be found, in the online version, at https://doi.org/10.31083/RCM39689.

## References

[1] Sabik JF, Mehaffey JH, Badhwar V, Ruel M, Myers PO, Sandner S (2024). Multiarterial vs Single-Arterial Coronary Surgery: 10-Year Follow-up of 1 Million Patients. *The Annals of Thoracic Surgery*.

[2] Hamilton GW, Raman J, Moten S, Matalanis G, Rosalion A, Dimagli A (2023). Radial artery vs. internal thoracic artery or saphenous vein grafts: 15-year results of the RAPCO trials. *European Heart Journal*.

[3] Gaudino M, Benedetto U, Fremes S, Ballman K, Biondi-Zoccai G, Sedrakyan A (2020). Association of Radial Artery Graft vs Saphenous Vein Graft with Long-term Cardiovascular Outcomes among Patients Undergoing Coronary Artery Bypass Grafting: A Systematic Review and Meta-analysis. *JAMA*.

[4] Taggart DP, Altman DG, Gray AM, Lees B, Nugara F, Yu LM (2010). Randomized trial to compare bilateral vs. single internal mammary coronary artery bypass grafting: 1-year results of the Arterial Revascularisation Trial (ART). *European Heart Journal*.

[5] Chan J, Dimagli A, Dong T, Fudulu DP, Sinha S, Angelini GD (2022). Trend and factors associated with multiple arterial revascularization in coronary artery bypass grafting in the UK. *European journal of cardio-thoracic surgery: official journal of the European Association for Cardio-thoracic Surgery*.

[6] Rotar EP, Scott EJ, Hawkins RB, Mehaffey JH, Strobel RJ, Charles EJ (2023). Changes in Controllable Coronary Artery Bypass Grafting Practice for White and Black Americans. *The Annals of Thoracic Surgery*.

[7] Lawton JS, Tamis-Holland JE, Bangalore S, Bates ER, Beckie TM, Writing Committee Members (2022). 2021 ACC/AHA/SCAI Guideline for Coronary Artery Revascularization: A Report of the American College of Cardiology/American Heart Association Joint Committee on Clinical Practice Guidelines. *Journal of the American College of Cardiology*.

[8] Hamilton GW, Theuerle J, Chye D, Bhaskar J, Seevanayagam S, Johns H (2024). Graft Patency and Clinical Outcomes in Patients With Radial Artery Grafts Previously Instrumented for Cardiac Catheterization. *Circulation. Cardiovascular Interventions*.

[9] Schwann TA, Zacharias A, Riordan CJ, Durham SJ, Shah AS, Habib RH (2009). Sequential Radial Artery Grafts for Multivessel Coronary Artery Bypass Graft Surgery: 10-Year Survival and Angiography Results. *The Annals of Thoracic Surgery*.

[10] van Son JAM, Smedts F, Vincent JG, van Lier HJJ, Kubat K (1990). Comparative anatomic studies of various arterial conduits for myocardial revascularization. *The Journal of Thoracic and Cardiovascular Surgery*.

[11] Nakajima H, Kobayashi J, Tagusari O, Bando K, Niwaya K, Kitamura S (2006). Functional Angiographic Evaluation of Individual, Sequential, and Composite Arterial Grafts. *The Annals of Thoracic Surgery*.

[12] Hu Z, Wang C, Yuan X, Zhang S, Chen S, Hou Z (2023). Potential of Quantitative Flow Ratio for Selecting Target Vessels for Radial Artery Grafting: a Retrospective Observational Study. *Circulation*.

[13] Tonino PAL, De Bruyne B, Pijls NHJ, Siebert U, Ikeno F, van’ t Veer M (2009). Fractional flow reserve versus angiography for guiding percutaneous coronary intervention. *The New England Journal of Medicine*.

[14] Acar C, Jebara VA, Portoghese M, Beyssen B, Pagny JY, Grare P (1992). Revival of the radial artery for coronary artery bypass grafting. *The Annals of Thoracic Surgery*.

[15] Buxton BF, Hayward PA, Raman J, Moten SC, Rosalion A, Gordon I (2020). Long-Term Results of the RAPCO Trials. *Circulation*.

[16] Saadat S, Habib R, Engoren M, Mentz G, Gaudino M, Engelman DT (2023). Multiarterial Coronary Artery Bypass Grafting Practice Patterns in the United States: Analysis of the Society of Thoracic Surgeons Adult Cardiac Surgery Database. *The Annals of Thoracic Surgery*.

[17] Nakajima H, Kobayashi J, Toda K, Fujita T, Shimahara Y, Kasahara Y (2012). Angiographic evaluation of flow distribution in sequential and composite arterial grafts for three vessel disease. *European Journal of Cardio-Thoracic Surgery*.

[18] Moshkovitz Y, Raanani E (2016). The art of saphenous vein grafting and patency maintenance. *The Journal of Thoracic and Cardiovascular Surgery*.

[19] Gaudino M, Puskas JD, Di Franco A, Ohmes LB, Iannaccone M, Barbero U (2017). Three Arterial Grafts Improve Late Survival: A Meta-Analysis of Propensity-Matched Studies. *Circulation*.

[20] Schwann TA, El Hage Sleiman AKM, Yammine MB, Tranbaugh RF, Engoren M, Bonnell MR (2018). The Incremental Value of Three or more Arterial Grafts in CABG: the Effect of Native Vessel Disease. *The Annals of Thoracic Surgery*.

[21] Charney R, Cohen M (1993). The role of the coronary collateral circulation in limiting myocardial ischemia and infarct size. *American Heart Journal*.

