# Supplementary material for: Comparison of Patency Between Sequential and Individual Radial Artery Anastomoses in Coronary Artery Bypass Grafting
Source: Rev Cardiovasc Med. 2025 Nov 12;26(11):39689. doi: 10.31083/RCM39689 (PMC12680978; doi:10.31083/RCM39689)
Supplement: Supplementary file 1 [file 2153-8174-26-11-39689-s1.docx]

**Supplements**

**Supplementary Table 1 The percentages of imaging follow-up at each time frame**

| **Time Frame** | **Patients**  **Follow-up percentage** |
| --- | --- |
| 3 months | 34.7% (95/274) |
| 12 months | 61.7% (169/274) |
| 36 months and beyond | 52.9% (145/274) |

**Supplementary Table 2 Univariate and multivariate analysis of risk factors for MACCEs**

| **Variable** | **HR (univariate)** | | ***P*-value** | | | **aHR (multivariate)** | | ***P*-value** |
| --- | --- | --- | --- | --- | --- | --- | --- | --- |
| Sequential graft | | 0.39(0.05-3.02) | | 0.364 | 0.14(0.01-1.63) | | | 0.117 |
| QFR > 0.71 | | 1.17(0.34-4.01) | | 0.798 | 1.80(0.46-7.04) | | | 0.397 |
| Age > 60 years | | 3.72(1.11-12.43) | | 0.033 | 11.11(2.21-55.86) | | 0.003 | |
| Female | | 3.33(1.11-12.43) | | 0.260 | 2.81(0.25-31.51) | | 0.400 | |
| BMI | | 1.00(0.82-1.22) | | 0.980 | 1.06(0.82-1.38) | | 0.666 | |
| EF < 50% | | 0.61(0.08-4.80) | | 0.637 | - | | - | |
| Diabetes | | 1.36(0.41-4.46) | | 0.612 | - | | - | |
| Hypertension | | 0.93(0.28-3.05) | | 0.906 | - | | - | |
| Hypercholesterolemia | | 0.66(0.41-3.09) | | 0.599 | - | | - | |
| PVD | | 2.01(0.26-15.76) | | 0.505 | - | | - | |
| Prior MI | | 1.13 (0.33–3.88) | | 0.842 | 2.35(0.48-11.58) | | 0.291 | |
| Smoking | | 0.32(0.10-1.06) | | 0.062 | - | | - | |
| On-pump surgery | | 0.90(0.27-3.01) | | 0.858 | 0.72(0.16-3.29) | | 0.670 | |
| Target on left system | | 0.55(0.15-2.07) | | 0.376 | 0.32(0.05-1.89) | | 0.208 | |

MACCEs, major adverse cardiac or cerebrovascular events; HR, hazard ratio; aHR, adjusted hazard ratio; QFR, quantitative flow ratio; BMI, body mass index; EF, ejection fraction; PVD, peripheral vascular disease;MI, myocardial infarction.

**Supplementary Fig. 1:** **An example of QFR computation in** **left circumflex branch.**


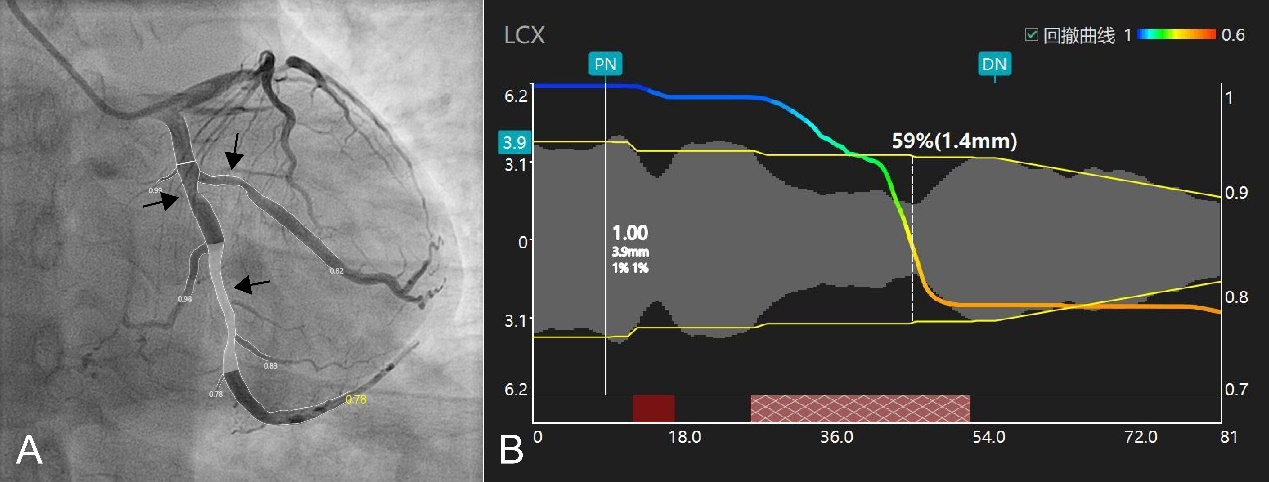


A, Three main lesions can be seen in angiogram (indicated by black arrows), and the lumen contour of left circumflex branch and its side branch was automatically delineated; B, The vessel diameter graph and the virtual QFR pull-back curve. PN indicates proximal normal reference diameter; and DN, distal normal reference diameter. The QFR of obtuse marginal branches 1 and 2 were computed as 0.82 and 0.78, respectively.

**Supplementary Fig. 2: The outcome definition of sequential graft.**


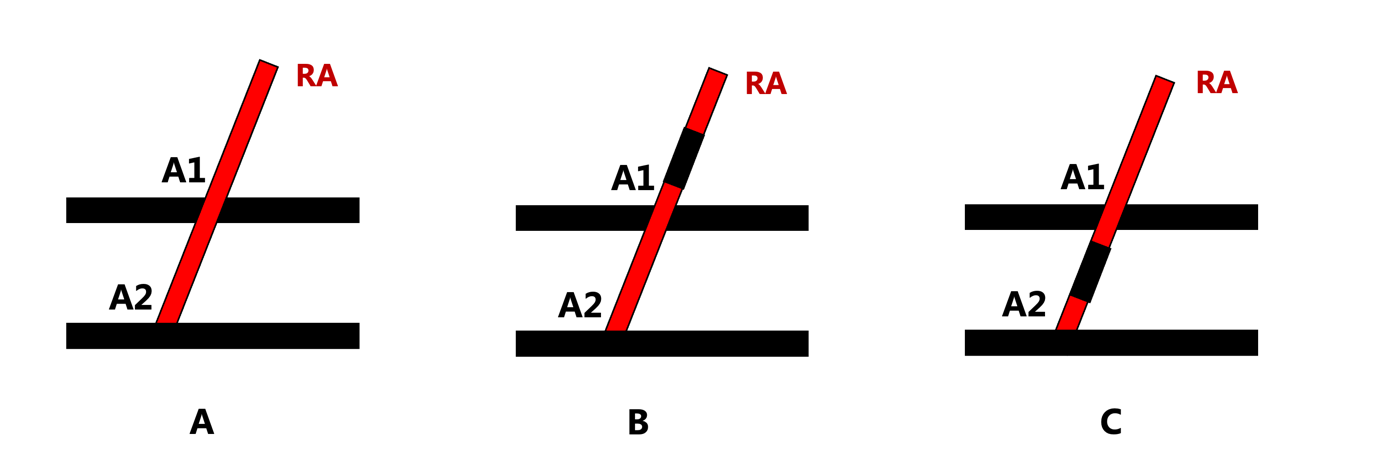


A: patent; B&C: occluded.

**Supplementary Fig. 3 Subgroup analyses and interaction terms for the graft patency.**

**
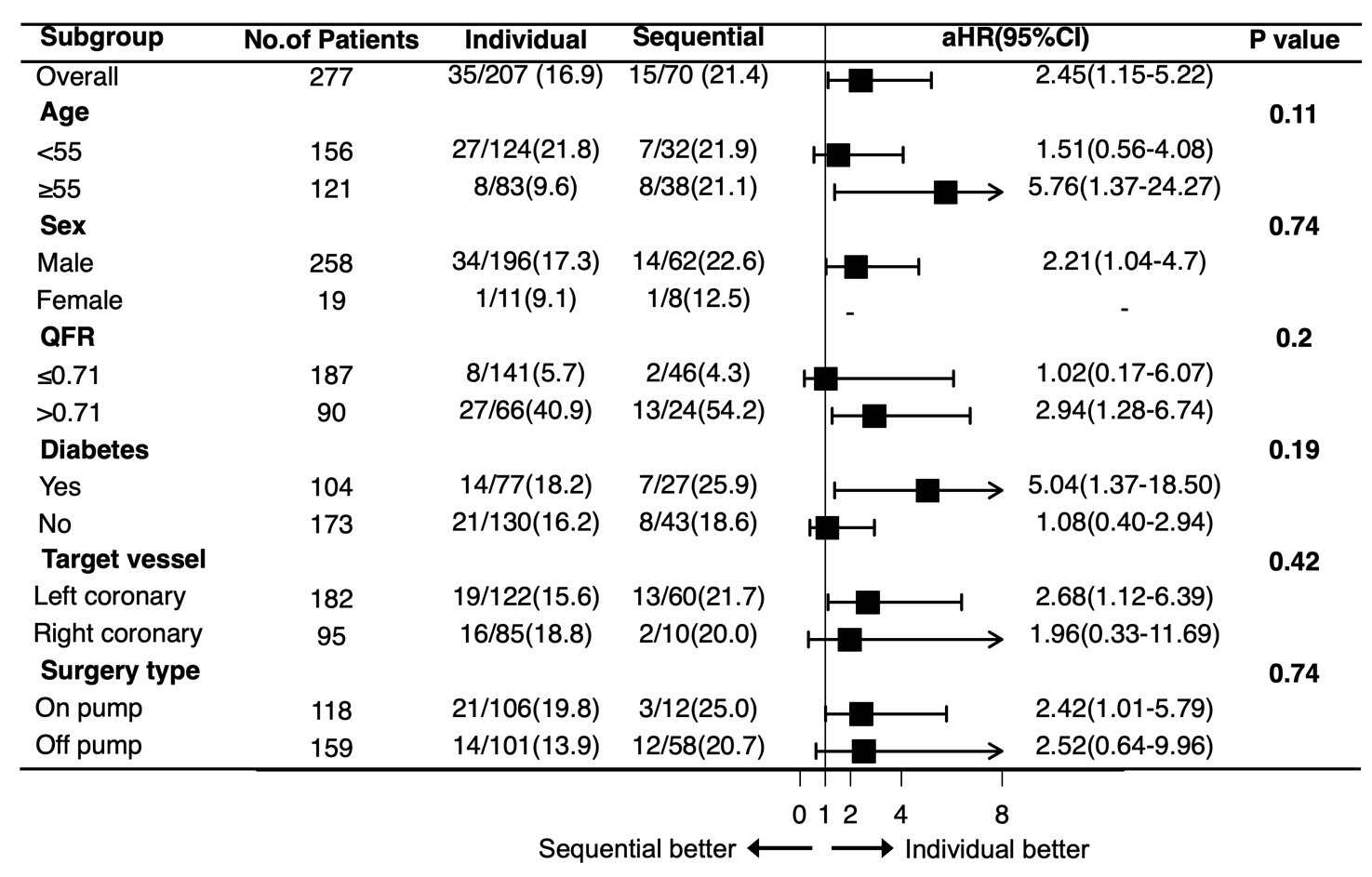
**

The P values given are the P values for the interaction-term analyses. It was unavailable to calculate the hazard ratio in the female subgroup because of the limited sample size of female patients.

**Supplementary Fig. 4: The Kaplan-Meier curve of patient outcomes.**


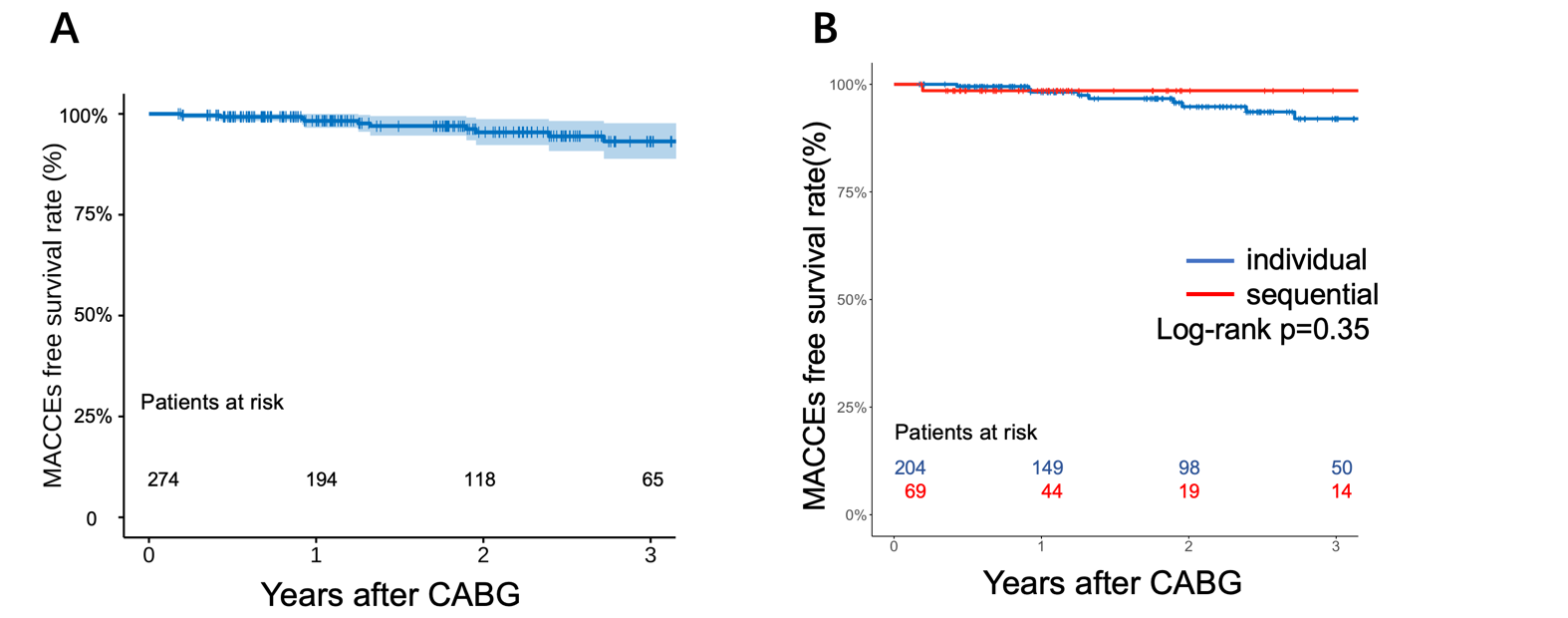


A: The Kaplan-Meier curve of MACCEs free survival of total population. B: Comparison of cumulative MACCEs free survival between individual and sequential groups. 1 patient who both received individual and sequential RA grafts was excluded.

MACCEs indicates major adverse cardiac or cerebrovascular events of patients, including death from any cause, myocardial infarction, stroke, or repeat revascularization; CABG, coronary artery bypass grafting.
